# Visceral fat area and albumin based nutrition-related prognostic index model could better stratify the prognosis of diffuse large B-cell lymphoma in rituximab era

**DOI:** 10.3389/fnut.2022.981433

**Published:** 2022-09-08

**Authors:** Ziyuan Shen, Lingling Hu, Shuo Zhang, Qian Sun, Weidong Li, Dongmei Yan, Guoqi Cai, Wei Sang

**Affiliations:** ^1^Department of Epidemiology and Biostatistics, School of Public Health, Anhui Medical University, Hefei, China; ^2^Department of Hematology, Affiliated Hospital of Xuzhou Medical University, Xuzhou, China; ^3^Department of Radiology, Affiliated Hospital of Xuzhou Medical University, Xuzhou, China

**Keywords:** DLBCL, visceral fat area, albumin, nutritional indices, prognosis

## Abstract

**Background:**

Diffuse large B-cell lymphoma (DLBCL) is a heterogeneous disease and the existing prognosis systems based on clinical variables are difficult to stratify patients accurately. Nutritional indices play a meaningful role in prognosis of solid tumors, whereas the effect on DLBCL is still equivocal. This retrospective study aimed to develop a novel model based on nutritional indices and other clinical variables to accurately differentiate the prognosis of DLBCL.

**Methods:**

A total of 129 patients pathologically diagnosed with DLBCL in Affiliated Hospital of Xuzhou Medical University from 2014 to 2018 were retrospectively recruited. The total fat area (TFA), visceral fat area (VFA) and subcutaneous fat area (SFA) at the third lumbar vertebra level spine were obtained by computed tomography (CT) to assess the effect of nutritional status on the prognosis of DLBCL. Principal component analysis was used to reduce the dimension of nutritional indices, and continuous variables were evaluated according to X-Tile and Restricted cubic spline. Univariable and multivariable Cox proportional hazard analyses were performed on potential variables. Kaplan-Meier method was utilized to evaluate survival probabilities and the differences between groups were assessed by log-rank test.

**Results:**

X-Tile analysis divided VFA and albumin into two and three groups when applying 114.7 cm^2^ of VFA, 38.3 and 42.4 g/L of albumin as the optimal cut-off points, respectively. The final scoring model of nutrition-related prognostic index (NPI) comprised four independent prognostic variables. The C-index of the final model was 0.823 [95% *CI* (0.749~0.897)] by bootstrap resampling. Finally, a maximum score of 6 points was obtained. Compared with IPI, NCCN-IPI and GELTAMO-IPI, NPI showed better accuracy in discerning the prognostic risk of patients.

**Conclusion:**

VFA and albumin were associated with the prognosis of DLBCL, and the NPI model based on nutritional indices could better stratify the prognosis of DLBCL.

## Introduction

Diffuse large B-cell lymphoma (DLBCL) is an aggressive lymphoma, accounting for about 40% of non-Hodgkin lymphoma (NHL) (1). High heterogeneity, genetic diversities, immune markers, nutritional indices and clinical variables significantly affect the prognosis of DLBCL. The majority of DLBCL patients can be cured with rituximab based immunochemotherapy and optimization of frontline therapy remains an important goal. Previous studies have shown that age, Ki-67, β_2_-microglobulin, lactate dehydrogenase (LDH), and other clinical variables are independent prognostic factors of DLBCL (2–5). In the past decades, clinical variables based prognostic models such as International Prognostic Index (IPI) and NCCN-IPI were widely adopted to evaluate the prognosis of DLBCL with certain limitations (2, 6). However, few studies were focused on the prognosis of DLBCL patients according to their nutritional status and body composition.

Nutritional status closely correlates with various metabolic processes, which also affects quality of life, therapeutic regimen and prognosis in cancer patients (7). Many nutrition indices-based scoring systems such as cachexia score (CASCO), Glasgow prognostic score (GPS) and prognostic nutritional index (PNI) have been used to stratify the prognosis of solid tumors (8–10). Sungwoo Park et.al revealed that DLBCL patients in low-PNI group showed more frequent treatment-related toxicities and mortality (11). A systematic review showed that body mass index (BMI) was associated with the risk of prognosis in patients with DLBCL and the summary relative risk (RR) per 5 kg/m^2^ increment of BMI was 1.11 (95% *CI*: 1.05~1.16) for DLBCL (12). McMillan et al. confirmed that low albumin concentrations correlated with poor survival in patients with gastric, rectal, and esophageal squamous cell cancers (13–15). In recent years, human body composition analysis has become an indicator of nutrition monitoring, and it can reflect human health level and nutritional status. Computed tomography (CT), the gold standard for quantitative measurement of regional human adipose tissue with the advantages of accurate positioning and quantification, simple operation and high-density resolution, provides a reference for regional adipose tissue quantification. Grillot et al. demonstrated that sarcopenia and visceral obesity were associated with adverse outcomes in patients with Crohn's disease by CT (16). Excessive area of subcutaneous fat in the abdomen can secrete a large amount of chemicals, resulting in metabolic changes, thus increasing the burden of the heart and the risk of other cancers. Visceral fat acts as a large endocrine gland, excreting cytokines and adipocytes, leading to insulin resistance and proinflammatory states (17). Studies have shown that visceral obesity may be a better predictor than BMI of morbidity and mortality associated with cardiovascular disease and type 2 diabetes (17–20). However, the effect of visceral obesity on the prognosis of DLBCL patients is still unclear and worth exploring.

In this retrospective study, we obtained total fat area (TFA), visceral fat area (VFA) and subcutaneous fat area (SFA) data at the section of the third lumbar by CT. We aimed to evaluate the effect of nutritional status on the prognosis of DLBCL by measuring patients' nutrition indices and introduced other clinical variables to develop a novel scoring prognostic model.

## Materials and methods

### Patients

All enrolled hospitalized patients were pathologically diagnosed as DLBCL by at least two pathologists from 2014 to 2018 in the Affiliated Hospital of Xuzhou Medical University. Study approval was obtained from the independent Ethics Committees of each participating center in HHLWG and met Helsinki Declaration. Informed consent was obtained from each patient. At admission, the following variables were considered: age, gender, Ann Arbor stage, LDH, β_2_-microglobulin (β_2_-MG), B symptoms, ECOG PS, cell of origin (COO), and other newer interesting prognostic factors, such as red blood cell count (RBC), white blood cell count (WBC) and lymphocyte count (LYC).

Inclusion criteria: (1) Clear pathological diagnosis; (2) Rituximab and doxorubicin based immunochemotherapy; (3) Complete clinical and CT scanning data. Exclusion criteria: (1) Patients with other malignant tumors; (2) Patients with special types of lymphoma (primary central nervous system lymphoma, primary mediastinal DLBCL, transformed DLBCL). Follow-up was conducted through reviewing in-patient medical records and making phone calls. Overall survival (OS) was calculated as the interval between the time of diagnosis and death from any cause or the last follow-up.

Fluorescence *in situ* hybridization (FISH) analysis for MYC, BCL-6, and BCL-2 was performed in all cases using the LSI MYC or BCL-6 dual-color break-apart and LSI IGH@BCL-2 dual-color, dual fusion probes (Abbott Laboratories, Des Plaines, IL, USA). All cases in this study with MYC, BCL-2, and BCL-6 rearrangements had abnormal signals present in >20% of all nuclei assessed. The operation was carried out according to the probe instructions described previously. By immunohistochemical (IHC), patients were divided into germinal center B cell (GCB) and Non-GCB groups (21).

### Assessment of body composition by CT scan

**Figure 1:**
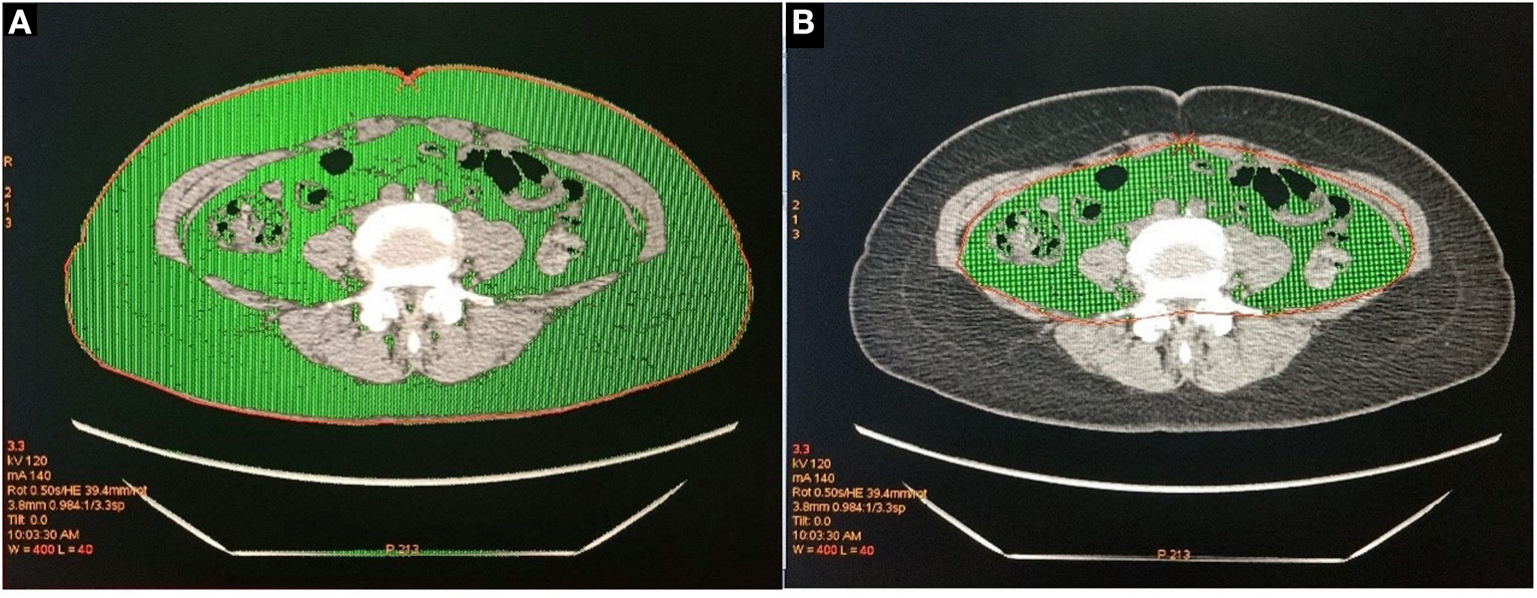
**(A)** The green part is the total abdominal fat area (TFA). **(B)** The green part is visceral fat area (VFA).

This research used the tomography/computed tomography (PET/CT) examination to obtain CT images. All patients, placed in a supine position with both upper limbs raised above the head, underwent whole-body CT examination using discovery PET / CT elite scanner (GE Healthcare, Milwaukee, Wisconsin, USA) and light speed 128 slice spiral. After the completion of the CT localization image, CT scanning was performed and the corresponding data were collected. The scanning range was from the cranial apex to the middle part of the thigh. CT scanning parameters: 120 kV, 180 mA, rotation time 0.5 s/r, pitch 1.375, layer thickness 3.75 mm, layer spacing 3.25 mm. After scanning, the whole-body CT image was transmitted to GE Advantage workstations (GEAW) 4.5 post-processing workstation, the image was positioned to the level of navel, and the “X-Sect” mode was started to draw the total area along the abdominal skin contour, and then the abdominal area was drawn along the inner edge of abdominal wall muscle tissue, and the fat attenuation range (−190 ~ −30 Hounsfield units) was adjusted. The color part is the fat area (Figure 1), and then the post-processing workstation calculates the TFA and VFA. SFA was calculated by subtracting VFA from TFA. VFA, TFA, SFA were computed for each image in cm^2^.

## Statistical analysis

Principal component analysis (PCA) is a mathematical algorithm that reduces the dimensionality of a data while retaining as much as possible the variation in the data. The samples can then be plotted to visually evaluate the similarities and differences between the samples and determine whether the samples can be grouped (22). Continuous variables were transformed into categorical variables by X-Tile program (23) or restricted cubic spline (RCS) (24). Pearson's correlation analysis was used to analyze the correlation between nutritional markers and survival time. Univariable analysis was used to select variables, with *P* < 0.1 considered statistically significant. Multivariable analysis was performed for predictive prognostic variables. The best prediction variable set was obtained by both stepwise regression and Akaike Information Criteria (AIC) (25) was used to evaluate the optimal model. The Harrell's concordance index of the model was calculated by bootstrap method according to the regression results. The procedure was repeated 1,000 times.

Decision curve analysis (DCA) is a novel method for evaluating diagnostic tests, prediction models, and molecular markers to determine whether a model or one of several models is needed (26, 27). In order to evaluate the effect of nutritional factors on the prognosis of DLBCL, we established a nutrition-related prognosis index (NPI) model based on the separate analysis of PCA (Model 1) and the accurate cut-off points of continuous variables (Model 2), and the performance of the two models was compared using DCA. Statistical analysis was conducted with IBM SPSS Statistics (version 19.0) and R software (version 3.6.1; http://www.Rproject.org).

## Results

### Clinical characteristics

The follow-up deadline was May 1, 2020, and the median follow-up time was 19.1 months. At the end of follow-up, a total of 43 (33.33%) deaths occurred. Median age at diagnosis was 61 years (range: 21–87), of whom 62 were males. Ann Arbor stage I, II, III, IV period accounted for 26.4, 30.2, 22.5 and 20.9%, respectively. Only 23.26% of patients had B symptoms. Patient characteristics were shown in Table 1.

**Table 1:** General features of DLBCL patients.

**Characteristics**	***n* (%)**
Age	
<60	63 (48.84)
≥60	66 (51.16)
Gender	
Male	62 (48.06)
Female	67 (51.94)
Ann arbor stage	
I-II	73 (56.58)
III-IV	56 (43.42)
ECOG score	
0–1	115 (89.15)
≥2	14 (10.85)
Bulky disease	
Absence	116 (89.90)
Presence	13 (10.10)
B symptoms	
Absence	99 (76.74)
Presence	30 (23.26)
Bone marrow involvement	
Absence	104 (80.60)
Presence	25 (19.40)
Hemoglobin(g/L)	
<130	71 (55.00)
≥130	58 (45.00)
LDH	
Normal	65 (50.40)
Elevated	64 (49.60)
Albumin [median (IQR)]	42.80 (38.80–45.95)
BMI [median (IQR)]	24.00 (21.80–26.10)
Extranodal involvement	
Yes	11 (8.50)
No	118 (91.50)
IPI score	
0~2	87 (67.44)
3~5	42 (32.56)

LDH, lactate dehydrogenase; BMI, body mass index; IPI, International prognostic index.

### Analysis of nutrition-based indices

**Figure 2:**
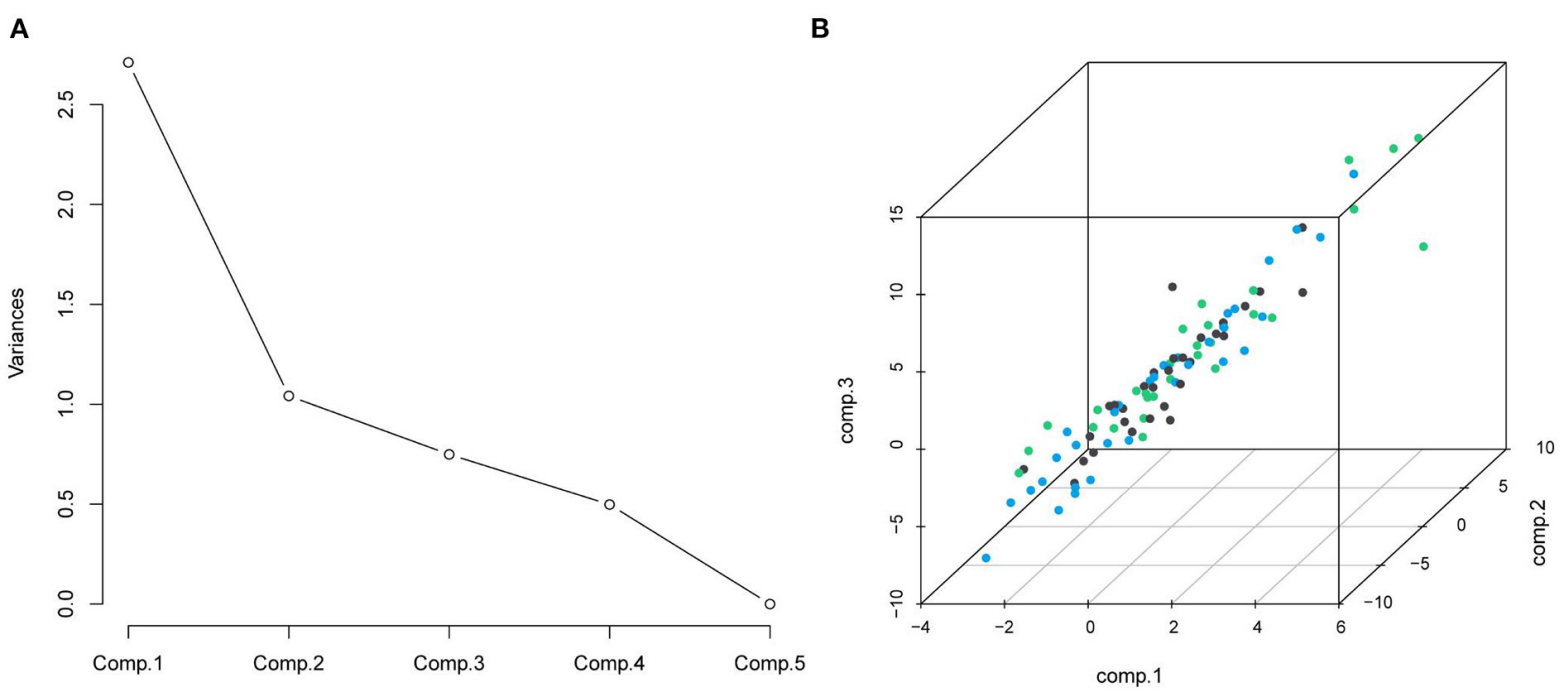
**(A)** Scree plot from principal components analysis of nutrition elements; the five components were: TFA, VFA, SFA, BMI, and albumin; **(B)** the distribution of the three principal components.

**Figure 3:**
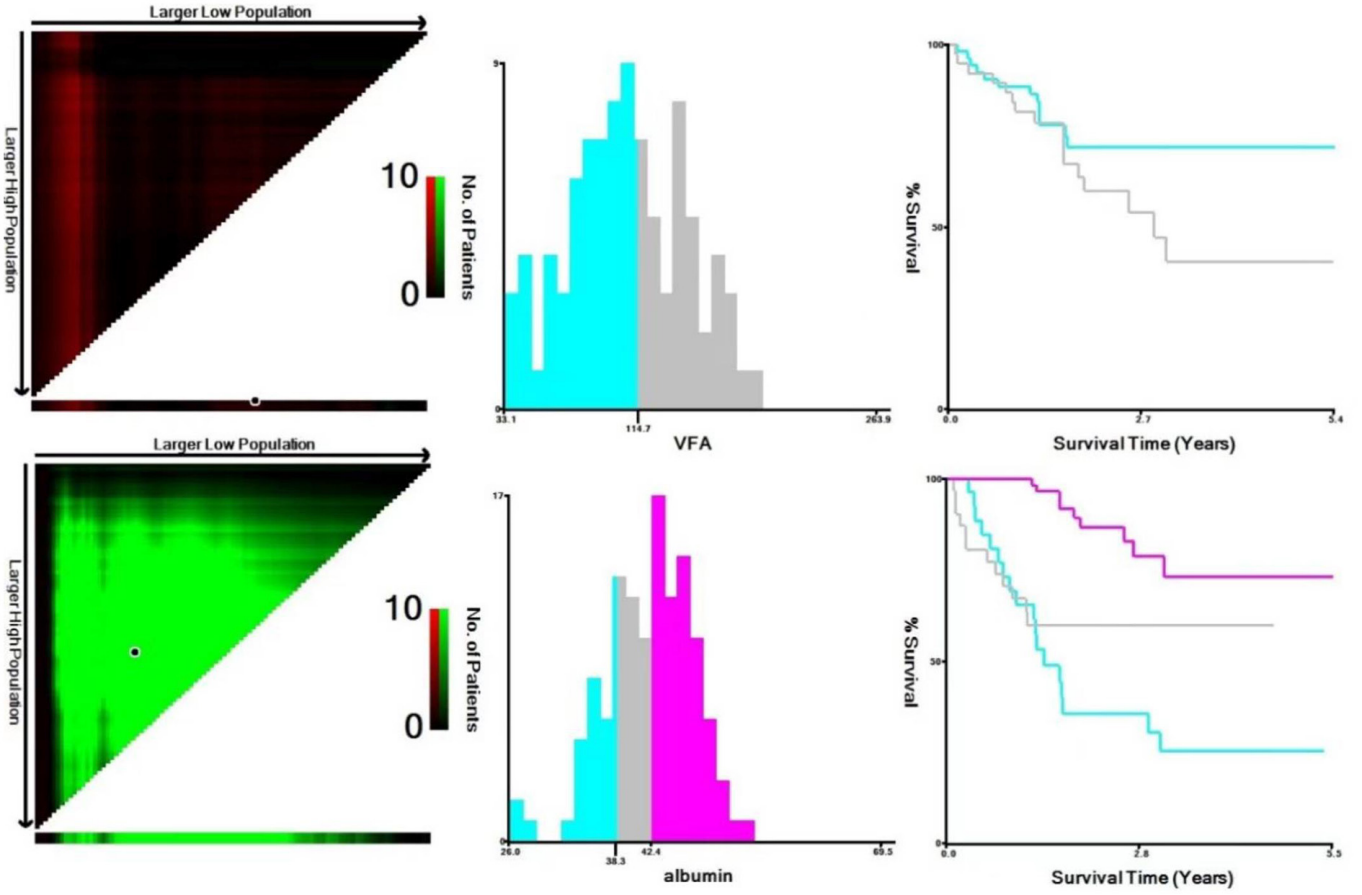
Analysis of VFA and albumin using X-Tile program. The black circles highlight the optimal cutoff values which were presented in histograms.

The impact of nutritional status expressed by nutritional variables (TFA, VFA, SFA, BMI and albumin) on the prognosis of DLBCL patients was explored by two statistical methods. First of all, correlation analysis showed that all markers above were correlated with survival. Furthermore, albumin had the strongest correlation with survival time (r = 0.6, *P* < 0.001). PCA extracted three components from five nutritional markers, among which the characteristic values of the first and second principal components were both greater than 1, and the contribution values of the first, second and third principal components were 90.04% (Figure 2). The relevant scores of each patient were calculated, and the scores were evaluated and converted using X-Tile program. Second, data type conversion was performed for the five nutritional markers. By using X-Tile program, TFA, VFA, SFA and BMI were divided into high and low groups, and albumin was divided into three groups. The maximum chi-square point of 5.163 was reached when applying SFA = 145.3 cm^2^ as the optimal cut-off point. Similarly, the optimal cut-off points of TFA and VFA were 210.7 cm^2^ and 114.7 cm^2^, respectively. The optimal cut-off point for BMI was 27.3 kg/m^2^ (*P* < 0.05). The optimal thresholds of albumin were 38.3 g/L and 42.4 g/L for survival prediction in DLBCL patients (*P* < 0.001, Figure 3).

### Cut-off points identification of continuous variables

Continuous variables included in this study, registered at the time of diagnosis, were age, LDH, β_2_-microglobulin, globulin, RBC, WBC, LYC, hemoglobin and platelet. The continuous variables were processed separately by X-Tile program, RCS and laboratory reference range. The results showed that there was a non-linear correlation between age and death risk (*P* < 0.001). The X-Tile program divided age into three groups. By comparing the results of RCS and X-Tile, two cut-off points could more accurately indicate the relationship between age and survival of DLBCL (*P* < 0.001, X-Tile; *P* = 0.011, RCS). Similarly, LDH, Ki-67, RBC, β2-microglobulin and globulin were grouped by X-Tile program. WBC was grouped using the RCS. The normal reference range (130–175g/L) could better reflect the relationship between hemoglobin and survival outcome.

### Univariable and multivariate analysis of DLBCL patients

Based on the two statistical analysis methods, we constructed two models related to DLBCL prognosis. The results of univariable analysis of Model 1 revealed that β2-microglobulin, globulin, Ann Arbor stage, age, Ki-67, hemoglobin, RBC, significantly affected OS (*P* < 0.05). β2-microglobulin appeared to be a stronger predictor (*P* < 0.001). The results of univariable analysis of variable extracted by PCA showed that it had an impact on the prognosis of DLBCL patients (*P* < 0.1). After iterative analysis of the multivariate model, the final prognostic indicator was composed of four factors (bone marrow involvement, β2-microglobulin, B symptoms, RBC, Table 2). Likewise, β2-microglobulin, globulin, Ann Arbor stage, age, Ki-67, hemoglobin, RBC, BMI significantly affected OS in univariable analysis of Model 2 (*P* < 0.05). Albumin, β2-microglobulin appeared to be stronger predictors (*P* < 0.001). After iterative analysis of the multivariate model, the final prognostic indicator was composed of four adverse factors (Table 2), which showed a better model fit (AIC = 140.02).

**Table 2:** Analysis of prognostic factors of OS in patients with DLBCL.

**Model 1**					**Model 2**				
**Univariable**		**Multivariable**			**Univariable**		**Multivariable**		
**Variables**	***P***	***HR***	***P***	***HR***	**Variables**	***P***	***HR***	***P***	***HR***
β2-MG	<0.001	3.766	0.019	3.992	VFA	0.062	1.893	0.036	2.662
Globulin	0.002	2.281			Albumin	<0.001	0.428	<0.001	0.204
Ann arbor stage	0.002	1.574			β2-MG	<0.001	3.766	0.007	5.036
Age	0.003	1.830			Globulin	0.002	2.281		
Ki-67	0.008	0.541			Ann Arbor stage	0.002	1.574		
Hemoglobin	0.008	0.391			Age	0.003	1.830		
RBC	0.029	0.494	0.025	0.314	Ki-67	0.008	0.541	0.013	0.421
WBC	0.059	1.869			hemoglobin	0.008	0.391		
PCA	0.098	0.519			RBC	0.029	0.494		
Cell of origin	0.144	1.665			BMI	0.033	0.115		
B symptoms	0.026	1.091	0.016	1.147	WBC	0.059	1.869		
Bone marrow involvement	0.019	1.104	0.004	5.585	TFA	0.123	0.560		

β2-MG, β2-microglobulin; RBC, red blood cell count; WBC, white blood cell count; PCA, Principal component analysis; VFA, visceral fat area; TFA, total fat area.

### Efficacy evaluation of nutrition-related prognosis index

**Figure 4:**
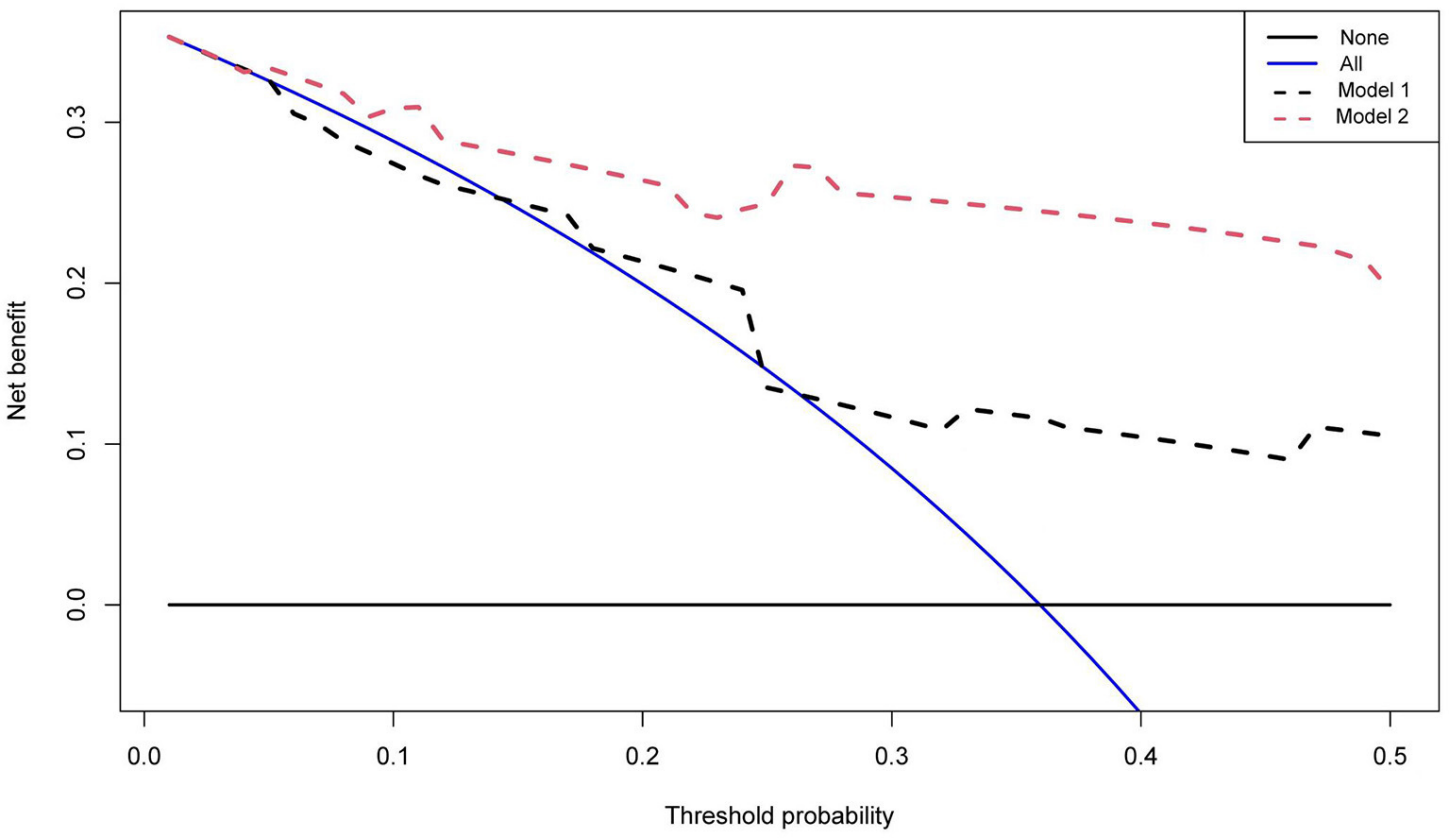
Decision curves of two multivariate models. The vertical axis represents the value of net benefit and the horizontal axis represents the threshold level (possible probabilistic pointcuts).

The net benefit of clinical predictors was plotted according to the threshold probability. Model 2 demonstrated an increased net benefit over Model 1 for predicting DLBCL patients (Figure 4). Afterwards, a prognostic model of DLBCL based on the exploration of nutritional indices and clinical usage was established (Table 3).

**Table 3:** Variables and scoring of Model 2.

**Variables**	**Score**
**VFA**	
<114.7	0
≥114.7	1
**Albumin**	
42.4–69.5	0
38.3–42.4	1
<38.3	2
**Ki-67**	
<0.6	0
0.6–0.8	1
≥0.8	2
**β2-MG**	
Normal	0
Elevated	1
**Maximum score**	6

VFA, visceral fat area; β2-MG, β2-microglobulin.

### Prognostic values of albumin with pathological patterns in DLBCL

**Figure 5:**
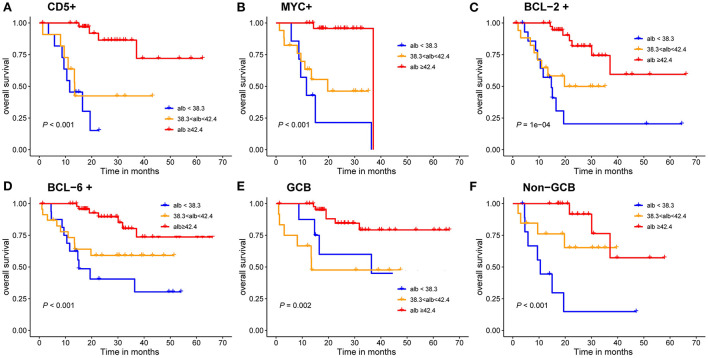
Kaplan-Meier survival curves of the DLBCL patients. **(A–D)** prognosis of different albumin levels in CD5 positive, c-Myc positive, BCL-2 positive and BCL-6 positive groups; **(E,F)** prognosis of different albumin levels in GCB and Non-GCB groups.

The results indicated that patients with albumin value <38.3 g/L had a poor prognosis. GCB patients accounted for 66.67% in all patients, and the results showed that albumin level was significant for the prognosis of GCB and Non-GCB patients (*P* < 0.005, Figures 5E, F). In addition, albumin level in both BCL-2 and BCL-6 negative groups had no effect on the prognosis of DLBCL patients (*P* = 0.170, *P* = 0.054), while different albumin level in BCL-2 and BCL-6 positive groups had influence on the prognosis (*P* < 0.001, Figures 5C, D). Similarly, different VFA level in c-Myc and BCL-2 negative groups had influence on the prognosis (*P* < 0.05, Figures 5A, B).

### Development and validation of nutrition-associated prognosis index

After exploration of nutritional indices and comparison by DCA, the current Model 2 used a maximum of 6 scoring points for categorized VFA, albumin, Ki-67 and β2-microglobulin (Table 3). This resulted in the establishment of four risk groups: low-risk (LR: 0–1points), low-intermediate risk (LIR: 2 points), high-intermediate risk (HIR: 3 points) and high-risk (HR: ≥ 4 points) with 2-year OS rates of 96.4, 70.9, 41.4 and 22.2%, respectively (Table 4). The C-index for the prediction model 2 was 0.823 [95% *CI* (0.749~0.897)] for validation.

**Table 4:** Two-year OS in patients using IPI, NCCN-IPI, GELTAMO-IPI scores.

	**IPI**	**NCCN-IPI**	**GELTAMO-IPI**	**Model 2**
LR	81	90	88	96.4
LIR	58	61	70	70.9
HIR	52	41.2	40	41.4
HR	45	-	20	22.2

IPI, International prognostic index; NCCN-IPI, National Comprehensive Cancer Network International prognostic index; GELTAMO-IPI, Grupo Español de Linfomas/Trasplante de Médula ósea International prognostic index; Model 2, the accurate cut-off points of continuous variables; LR, Low risk; LIR, Low intermediate risk; HIR, High intermediate risk; HR, High risk.

**Figure 6:**
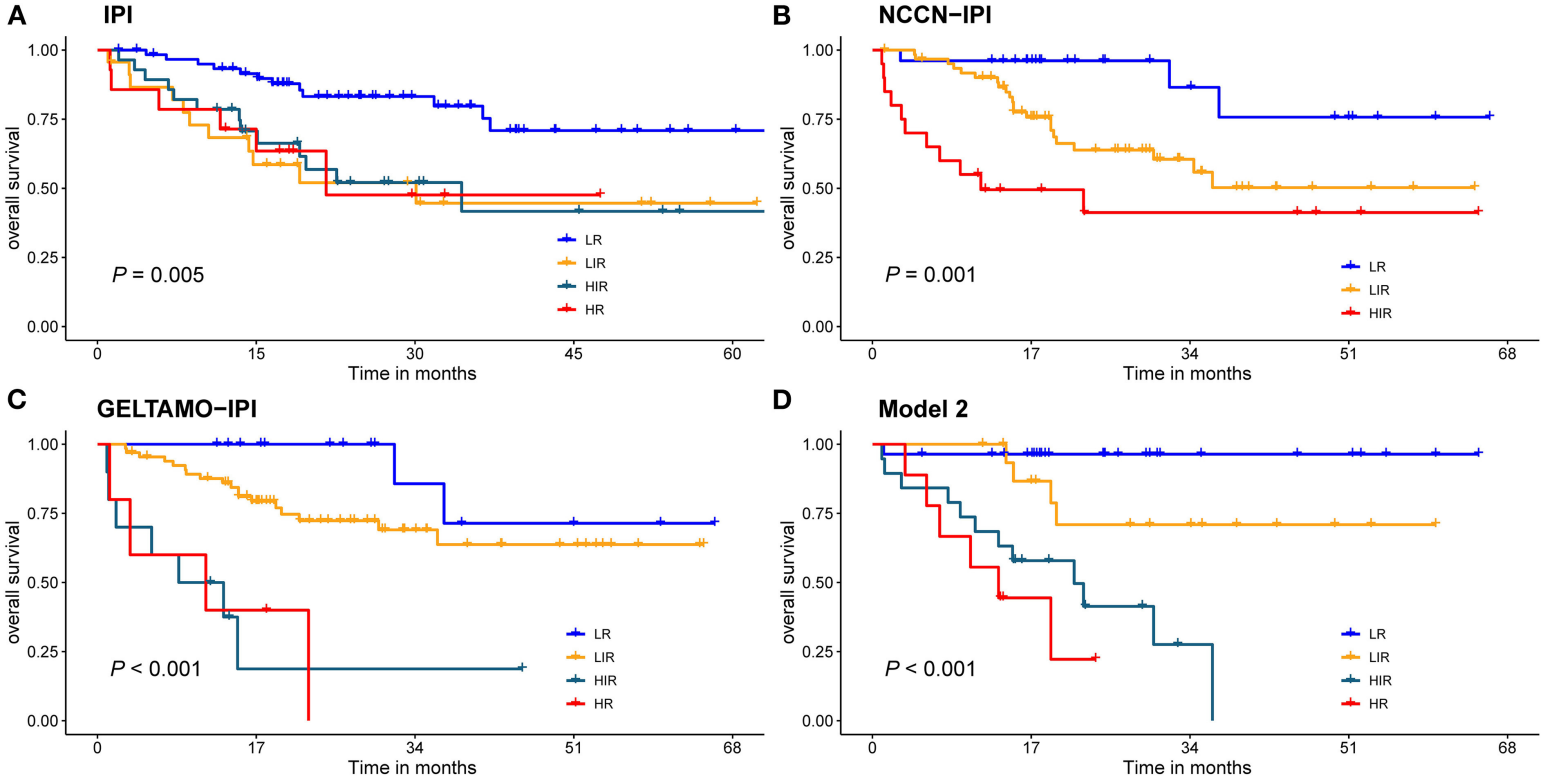
Risk groups according to stratification with the **(A)** IPI scores; **(B)** NCCN-IPI scores; **(C)** GELTAMO-IPI scores; **(D)** Model 2 scores. Global comparison *P* < 0.05.

All cases had complete data for the calculation of IPI, the 2-year OS rate for the LR, LIR, HIR and HR were 81, 58, 52 and 45%, respectively (Figure 6), and they were significantly different in the global (*P* < 0.010). Nevertheless, the IPI failed to distinguish LIR and HIR patients (*P* = 0.770). The NCCN-IPI separated patients into three risk groups and there was a marked difference between groups (LR vs. LIR: *P* = 0.021, LIR vs. HR: *P* = 0.019). The GELTMAO-IPI(Grupo Español de Linfomas/Trasplante de Médula ósea International prognostic index) separated patients into four risk groups and there was also a marked difference between LIR and HIR (*P* < 0.001) but it failed to distinguish LR and LIR (*P* = 0.140), HIR and HR (*P* = 0.940). However, model 2 could overcome the defects of IPI to distinguish LIR and HIR (*P* < 0.050). In addition, compared with NCCN-IPI, our model made a more obvious distinction between LIR and HIR.

## Discussion

In this retrospective study of newly diagnosed DLBCL patients, we demonstrated that nutritional indices of VFA and albumin could be independent prognostic factors for patients with DLBCL. By introducing β_2_-microglobulin and Ki-67, a nutrition-related prognosis index (NPI) was developed for a more accurate stratification of patients.

About one third of tumors patients are with poor nutritional status. The nutritional index PNI was identified as a powerful prognostic predictor of OS and PFS in patients with extranodal natural killer/T cell lymphoma (28). Similarly, it also could be used to predict the response to treatment, OS and PFS in DLBCL patients and low PNI predicted poor outcome (29). Visceral fat is an important index to evaluate recessive obesity and excessive accumulation of abdominal fat can easily cause abnormal glucose metabolism, lead to diabetes, and increase the risk of heart disease, colorectal cancer and uterine cancer (30–32). In this study, X-Tile program divided VFA into two groups with the optimal cut-off point of 114.7 cm^2^, whereas as reported before in the literature, visceral obesity was defined as a VFA≥130 cm^2^ (33). Univariable analysis showed that when the cut-off point of VAF was 130 cm^2^, it could not predict the prognosis of patients in our study (*P* = 0.12). When VFA exceeded 114.7 cm^2^, the mortality risk of DLBCL increased by 2.66 times. Albumin is widely recognized as a biomarker of nutritional status and its predictive roles of morbidity following outcomes in certain tumors have been illustrated (34–36). Albumin levels were divided into three groups and the optimal thresholds of albumin were 38.3 g/L and 42.4 g/L for survival prediction in DLBCL patients in our study. We also used X-Tile program to explore the optimal cut-off point for SFA (145.3 cm^2^), TFA (210.7 cm^2^) and BMI (27.3 kg/m^2^). Univariable analysis results showed that BMI could be a prognostic factor for the prognosis DLBCL, while TFA had no significance (*P* = 0.123). Furthermore, BMI was observed to be not predictive in multivariable analysis.

Based on nutritional indices, Model 2 showed a higher net benefit over Model 1 for predicting patients with DLBCL by using DCA. In univariable analysis, we found that three nutritional markers (VFA, albumin and BMI) were associated with prognosis of DLBCL. β_2_-microglobulin has been a classic prognostic factor in the pre-rituximab (37) and post-rituximab eras (38, 39). According to multivariable Cox regression, the coefficients of each risk factor were calculated, and the basic risk reference value was determined, then the score of each risk factor was calculated by scoring distance (40). The final model included four independent variables: VFA, albumin, Ki-67, and β_2_-microglobulin. The C-index was 0.823 between the predicted outcome and the real outcome of the model. NPI divides DLBCL patients into four groups with 2-year OS rates of 96.4, 70.9, 41.4 and 22.2%.

Previous studies have shown that different pathologic immunophenotypes influence the prognosis of DLBCL. In this study, we noticed that in different immunophenotypes, such as c-Myc and BCL-2 negative group, different levels of VFA could affect prognosis. Similarly, in different immunophenotypes, such as GCB, Non-GCB, BCL-2 and BCL-6 positive groups, different levels of albumin could affect the prognosis of DLBCL patients. The cut-off value of albumin was set at 3.5 g/dl in a previous study, while the lowest value of albumin in our study was 38.3 g/l, which may be due to the difference of ethnicity (41).

IPI has been the internationally recognized basis for the prognosis evaluation of DLBCL patients in the pre-rituximab era but it failed to accurately discern risk groups, especially high-risk patients due to the addition of rituximab to conventional CHOP regimens. On the basis of IPI, NCCN-IPI further refined the categorization of age and normalized LDH to better distinguish low-risk and high-risk subgroups (6). On the basis of NCCN-IPI, by adding β2-microglobulin, GELTAMO-IPI can better discriminate authentic high-risk group than NCCN-IPI and is not influenced by primary extranodal presentation or treatments of different intensity (5). The comparisons of the IPI, NCCN-IPI, GELTAMO-IPI and NPI were shown in Table 4 and Figure 6. Taking 3-year OS for example, IPI failed to discern authentic LR patients (3-y OS: 80%) but NCCN-IPI and GELTAMO-IPI showed better discrimination in identifying LR patients than IPI (3-y OS: 87%), while NPI showed a highest discernment in identifying LR patients (3-y OS: 90%). In addition, IPI did not distinguish between LIR and HIR groups. It is worth noting that in our study, NCCN-IPI failed to identify HR group, and GELTAMO-IPI failed to distinguish LR and LIR, HIR and HR groups. Although in NPI, there were no significant differences in HIR and HR, the survival curve was different.

In conclusion, the prognostic NPI model based on nutritional markers can accurately stratify low-risk patients and improve the prognostic assessment of patients with DLBCL. However, due to the retrospective study, small sample size and insufficient median follow-up time, this study has some limitations. In future prospective multicenter studies, the relationship between nutritional status and prognosis of DLBCL will be further explored.

## Data availability statement

The raw data supporting the conclusions of this article will be made available by the authors, without undue reservation.

## Author contributions

WS and GC contributed to conceptualization, supervision, and writing—review and editing. ZS and LH contributed to formal analysis and writing—original draft. SZ and QS contributed to acquisition of data. DY and WL contributed to conceptualization. All authors contributed to the article and approved the submitted version.

## Funding

This study was funded by the Natural Science Foundation of Jiangsu Province, Grant/Award Number BK20171181; Jiangsu Key Research and Development Project of Social Development, Grant/Award Number BE2019638; and Young Medical Talents of Jiangsu Science and Education Health Project, Grant/Award Number QNRC2016791.

## Conflict of interest

The authors declare that the research was conducted in the absence of any commercial or financial relationships that could be construed as a potential conflict of interest.

## Publisher's note

All claims expressed in this article are solely those of the authors and do not necessarily represent those of their affiliated organizations, or those of the publisher, the editors and the reviewers. Any product that may be evaluated in this article, or claim that may be made by its manufacturer, is not guaranteed or endorsed by the publisher.
